# Guarded Outcomes After Hip Hemiarthroplasty in Patients with Cerebral Palsy: Highlighting a Personalized Medicine Approach to Mitigate the Risk of Complications

**DOI:** 10.3390/jpm15060252

**Published:** 2025-06-15

**Authors:** Ahmed Nageeb Mahmoud, Nicholas R. Brule, Juan D. Bernate, Mark A. Seeley, Michael Suk, Daniel S. Horwitz

**Affiliations:** 1 Geisinger Musculoskeletal Institute, Geisinger Medical Center, Danville, PA 17822, USA; nbrule@geisinger.edu (N.R.B.); jbernate@geisinger.edu (J.D.B.); mseeley1@geisinger.edu (M.A.S.); msuk@geisinger.edu (M.S.); 2 Orthopedic Surgery Department, Faculty of Medicine, Ain Shams University, Cairo 11591, Egypt

**Keywords:** cerebral palsy, fracture, femoral neck, hip hemiarthroplasty, heterotopic ossification

## Abstract

**Background:** The effectiveness of hip hemiarthroplasty in managing femoral neck fractures in individuals with cerebral palsy has seldom been reported. **Objectives:** Given the complex neuromuscular issues associated with cerebral palsy (CP), this retrospective study aims to document the outcomes and characterize the complications of hip hemiarthroplasty for fractures of the femoral neck in a series of patients with CP, emphasizing the role of precision medicine in management. **Methods:** Six cases of hip hemiarthroplasty in six male patients with cerebral palsy and displaced femoral neck fractures have been reviewed in this study. The patients’ mean age at the time of surgery was 55.6 ± 14.1 years (range, 33–71). All the patients were independent indoor ambulators before their femoral neck fracture and had various medical comorbidities. Five patients had intellectual disabilities. **Results:** The mean clinical and radiographic follow-ups for the patients included in this series were 91.5 and 71.3 months, respectively. All the patients developed significant heterotopic ossification (HO) around the operated hip, which was observed as early as the second week postoperatively on radiographs. HO progressed throughout the follow-up for all the patients. One patient had an early postoperative dislocation with femoral stem loosening, which was managed by implant revision. Another patient had an acetabular protrusion, leading to the loss of their weight-bearing ability and mobility due to pain. Four patients were deceased at a mean of 86.5 months after the index surgery. **Conclusions:** After considering the preliminary evidence provided with this small case series, this study suggests the overall guarded outcomes of hip hemiarthroplasty in patients with CP. Given the 100% rate of heterotopic ossification, a precision medicine framework with consideration for HO prophylaxis may be recommended after hip hemiarthroplasty in patients with CP. It may also be reasonable to scrutinize a personalized risk assessment approach in this patient subset regarding decision making, surgical approach, and rehabilitation program. The clinical outcomes and the risks of complications following hemiarthroplasty should be sensibly presented to patients with cerebral palsy and their caregivers to achieve reasonable postoperative expectations.

## 1. Introduction

Cerebral palsy (CP) is caused by irreversible, non-progressive damage to the developing brain, leading to movement and postural defects [1]. It comprises various neuromotor impairment syndromes associated with progressive musculoskeletal deformities as the child advances in age [1,2]. From these musculoskeletal deformities, hip abnormalities, such as acetabular and proximal femoral dysplasia, hip subluxation, and dislocation, occur commonly in patients with CP [3,4]. These developmental hip problems are attributed to muscle imbalance, with the adductor, hip flexor, and medial hamstring muscle groups being relatively overactive compared with their antagonists [5], necessitating early clinical and radiographic monitoring to detect and manage before affecting the patient’s well-being and quality of life and becoming more challenging to intervene [6,7].

CP is the commonest cause of childhood disability in Western societies, with an incidence from 2 to 2.5 per 1000 live births [8]. Generally, individuals with cerebral palsy can typically expect to live between 30 and 70 years [9]. Those who have the highest life expectancies generally possess increased mobility, receive superior medical care and adaptive equipment, and have greater autonomy and independence [9]. In orthopedic care, the variation in neurological, cognitive, and functional presentations calls for a personalized treatment approach, where the interventions are customized according to the patient’s cerebral palsy (CP) subtype, weight-bearing status, intellectual level, and associated medical comorbidities.

Patients with cerebral palsy (CP) experience consistent neurological issues, such as abnormal muscle tone (primarily spasticity), chronic muscle weakness, balance problems, varying degrees of sensory loss [1,2,7], and mobility impairment [10]. In patients with cerebral palsy (CP), the mobility trends generally show a decline in walking ability over time, particularly during adulthood, with a significant portion experiencing deterioration in walking ability, either partial or complete, due to factors like chronic pain, fatigue, and changes in spasticity [10]. The other concerns for patients with CP may include intellectual impairment, seizures, visual and hearing impairment, and communication difficulty [2]. CP is often categorized based on the change in muscle tone: spastic, dyskinetic, or ataxic [11]. The other classifications are made according to the anatomical region involved and the severity of the problem [11,12].

Particularly, the management of hip conditions and the other sequelae of CP in pediatric and young adult patients varies based on the patient’s age, complaints, functional status, and patho-anatomy [13,14,15,16]. In patients with cerebral palsy (CP), the high incidence of osteoporosis is influenced by factors such as a reduced weight-bearing capacity, hormonal changes, insufficient calcium and vitamin D intake, and exposure to medications like anticonvulsants, all of which negatively impact bone health and renders patients with CP more likely to develop fractures, and particularly hip fractures, compared to patients without CP [17,18,19,20,21]. Concerning arthroplasty, total hip arthroplasty is acknowledged as a feasible option for adult, ambulatory patients with CP with degenerative hip disease [22,23,24,25], while hip hemiarthroplasty (HA) for adult patients with CP with displaced femoral neck fractures, only two case reports exist, reporting mainly on instability-related complications [26,27].

Precision medicine has been applied in several orthopedic applications to manage neuromuscular conditions, including preclinical genetic modelling [28] and the utilization of stem cell and molecular biology in management, ushering in the advent of innovative and precisely targeted therapeutic strategies and medications [29]. In the context of the therapeutic and preventive aspects of precision medicine, this study also tries to present an outcome-based precision medicine approach for such a patient setting.

With CP being considered one of the commonest causes of neglected femoral neck fractures worldwide [20,30,31,32], the aim of this retrospective study is to present the varied clinical and radiological outcomes of hip hemiarthroplasty in a series of patients with CP, all of whom were treated at the same institution. Based on the rarity of this patient setting, we try to propose an investigative perspective for future research rather than portraying generalizable conclusions.

## 2. Materials and Methods

### 2.1. Data Acquisition

We conducted a retrospective, institutional review board (IRB)-approved study to review the electronic medical records (EMRs) of our health systemto identify all the patients who underwent hip hemiarthroplasty (HA) from our institutional electronic medical database. Each HA case was independently reviewed, and the medical history of the patients was examined to identify the cases of cerebral palsy and exclude the cases with any other neuromuscular condition. This was confirmed by searching the EMRs and reviewing the results using the possible surgical procedural codes for hemiarthroplasty (CPT codes 27125 and 27236) and the diagnostic ICD-10 code for CP (G80). The study was conducted and reported in accordance with the STROBE (Strengthening the Reporting of Observational Studies in Epidemiology) guidelines. The completed STROBE checklist relevant to this study has been provided in the supplementary material (Table S1).

### 2.2. Patients

Two thousand, four hundred, and seventy-seven HA cases, performed in two thousand, three hundred, and forty-four patients, including cases of bilateral surgeries, have been reviewed for this study.. Out of them, six patients (0.25%) who had been diagnosed with cerebral palsy were identified and included in this study. All the patients (100%) were male, with an average age of 55.6 ± 14.1 years (range, 33–71) at the time of surgery. The left hip was involved in 4 out of 6 (66.7%) cases. At the time of surgery, the mean body mass index for all the patients was 22.15 ± 2.6 (range 17.2–25.2).

The associated neurological and medical comorbidities are shown in Table 1. All the individuals were able to walk independently indoors and sought medical attention after experiencing falls from a standing position. Three patients were uncertain about the exact timing of injury, and all the patients reported reduced activity levels within the few days before presentation.

### 2.3. Preoperative Assessment and Personalized Management Plan

**Figure 1:**
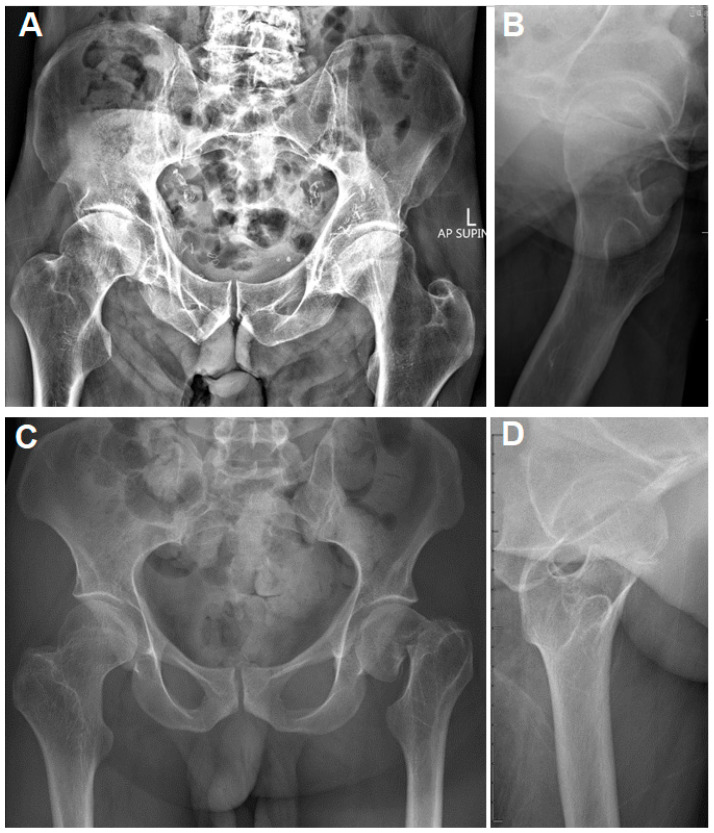
(**A**,**B**): Plain pelvis radiographs of a 71-year-old male with CP and a right valgus-impacted, ventrally tilted femoral neck fracture. The contralateral hip indicates an elevated femoral neck–shaft angle. (**C**,**D**): Plain pelvis radiographs of a 33-year-old male with CP and a left displaced femoral neck fracture. The contralateral femoral neck–shaft angle is increased as well.

In terms of CP classification, two patients had ataxic CP, two had spastic hemiplegia (one on the same side and one on the opposite side), and one had quadriparesis. The type of CP for one patient was not specified in the clinical records. Preoperative assessment of all the patients was conducted through physical examinations and radiographs, which revealed displaced, complete femoral neck fractures, along with an increased femoral neck–shaft angle (known as coxa valga) on the uninjured side (Figure 1) [19]. All the patients had a preoperative ASA score of 3.

The decision to proceed with hemiarthroplasty, rather than fixation or total hip arthroplasty (THA), for all 6 patients was based on the surgeon’s discretion. While THA may be the gold standard of management for patients with CP with femoral neck fractures, particularly neglected cases [20], its use on patients with CP, particularly those who have cognitive impairment or perform involuntary movements, carries an increased risk of dislocation, ranging from 0 to 28% [33,34,35,36]. In our retrospective study, the patients’ intellectual disabilities, indoor activity levels, history of frequent previous falls, and fracture displacement all influenced the decision to proceed with HA over another option. The management decision was made after a full discussion about the treatment options, risks, and benefits with the patients and their caregivers. Despite the relatively young age of the patients, including a 33-year-old patient, the presence of marked intellectual challenges and the limited indoor weight-bearing status, the severe radiographic displacement of the fracture, and the uncertainty regarding the exact timing of injury made HA a more practical decision, according to the treating physician.

### 2.4. Surgical Procedure

Given the presence of CP, the customized surgical plan aimed to maximize implant stability and perform any necessary soft tissue release if required. Surgeon selection was not standardized, but reflects real-life clinical practice. Five board-certified surgeons with extensive expertise in orthopedic trauma and/or adult reconstruction performed the surgeries using the anterolateral approach in four cases, while the posterior approach was utilized in two cases. The surgical approach was chosen according to the surgeon’s clinical verdict and experience. During the surgeries, precautions were taken to make a high femoral neck cut to preserve bone stock and restore the femoral offset in the presence of coxa valga. The surgeons also focused on restoring the natural hip mechanics, decreasing stem anteversion, and preserving the hip capsule for suitable capsular and muscle repair, which was carried out in all the cases. Out of the six patients, four were treated with cemented bipolar HA, one with cementless unipolar HA, and one with cementless bipolar HA. There were no significant intraoperative complications, and only one case required additional soft tissue release (iliopsoas release from the lesser trochanter). Intraoperative testing confirmed that all the components were stable within the tested ranges of motion. Additionally, intraoperative C-arm imaging was used to verify the concentric reduction in the femoral head into the acetabulum, femoral stem sizing, implant rotation, limb length, and the restoration of femoral offset.

After surgery, the patients were scheduled for regular clinical evaluations at 2 weeks and 6 weeks, followed by visits every six months. Weight bearing as tolerated began on the first day after surgery, with guidance from a physical therapist. The average hospital stay was 11 days (range: 2–30 days). Five patients were discharged to a nursing facility, and one patient was discharged home.

### 2.5. Statistical Analysis

Descriptive statistics were performed using Microsoft Excel (Microsoft Corporation, Redmond, WA, USA) to summarize the demographic and outcome data of the study participants. Continuous variables (such as patients’ age) are presented as means, standard deviations, and ranges, and descriptive variables (such as gender) are presented as percentages.

## 3. Results

This study includes six patients (six cases). The mean clinical and radiographic follow-ups were 91.5 ± 71.9 months (range, 26.1–178.6) and 71.3 ± 48.5 months (range, 16–154.2), respectively. Four patients expired at an average of 86.5 months after surgery due to causes unrelated to their surgery. The other two patients are still alive, with a mean duration of 116.2 months since surgery. At their final orthopedic follow-up, four patients were ambulatory using assistive devices, while two were wheelchair-bound. The mean calculated Parker Mobility Index for all the cases at their last clinical visit was 3.2 ± 1.94 (range 1–6).

**Figure 2:**
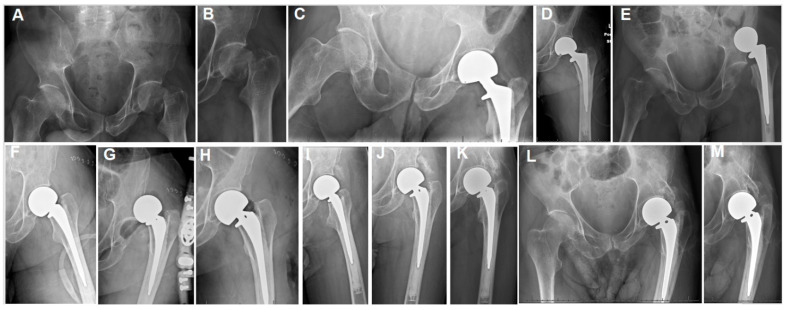
(**A**,**B**) The plain hip radiographs of a 55-year-old man who presented with a displaced left femoral neck fracture. (**C**,**D**) Postoperative plain hip radiographs taken immediately after cemented bipolar hemiarthroplasty. (**E**) One-week postoperative plain pelvic radiographs showing dislocated HA and stem loosening. (**F**) Plain hip AP radiographs after open revision. (**G**) A plain hip radiograph taken one day postoperatively showing re-dislocation. (**H**) Closed reduction was performed. (**I**) A plain hip radiograph taken at 4 weeks after the initial HA surgery, showing early HO lateral to the acetabular margin, along with stable HA components. (**J**) Eight-week and (**K**) 40-month postoperative plain radiographs showing progressive heterotopic ossification (HO). (**L**,**M**) Seventy-two-month postoperative radiographs showing class 3 acetabular and proximal femoral HO.

**Figure 3:**
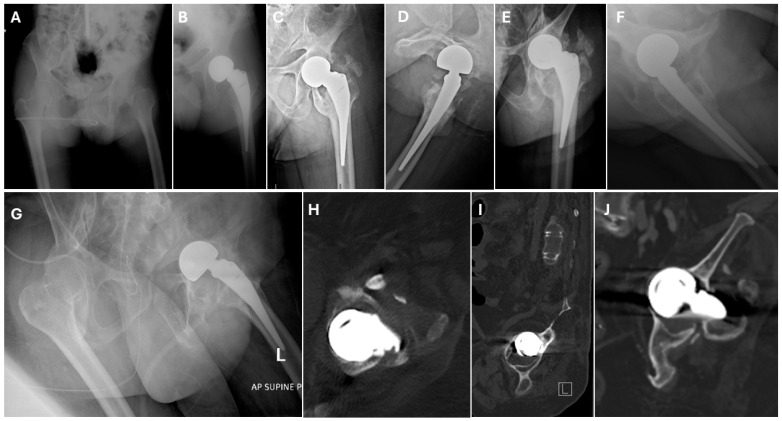
(**A**) The plain hip X-rays of a 47-year-old man with a displaced left femoral neck fracture. (**B**) Postoperative plain hip radiographs taken immediately following cementless bipolar hemiarthroplasty. (**C**,**D**) Twenty-two-month postoperative plain radiographs and CT scans of the pelvis, showing HO and radiographic acetabular erosion. (**E**,**F**) Postoperative plain hip radiographs at 61.6 months, showing HO and acetabular erosion progression. (**G**–**J**) X-rays and CT scan images taken approximately 177 months postoperatively (axial [H] and sagittal [I], and coronal [J]) showing an almost completely fused hip with class 4 HO and acetabular protrusion.

One patient (a cemented bipolar HA case) suffered an early postoperative dislocation (Figure 2) with femoral stem loosening, which was managed by implant revision with cemented HA again. The revision surgery was followed by a re-dislocation that was managed successfully with closed reduction and bracing. One patient had an acetabular protrusion (Figure 3), causing limited weight bearing due to pain. This was managed conservatively, but unfortunately required the patient to become wheelchair-bound.

**Figure 4:**
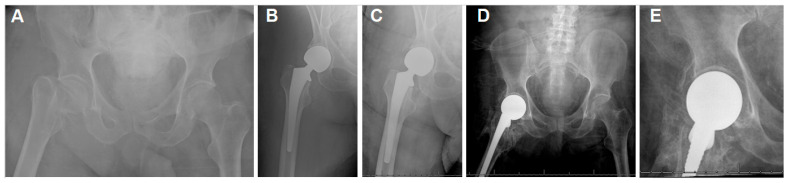
(**A**) The plain hip radiographs of a 68-year-old man who presented with a displaced right femoral neck fracture. (**B**) Postoperative plain hip radiograph taken immediately after cemented bipolar hemiarthroplasty. (**C**) Two-week postoperative plain pelvic radiograph showing stable HA and early HO. (**D**,**E**) Seventeen-month postoperative plain hip AP radiographs showing class 4 HO.

Notably, all the patients developed significant heterotopic ossification (HO) around the hip. HO occurred in the form of Brooker [28] class 4 (three patients had radiographic hip fusion—two of them were treated using the anterolateral technique, one using the posterior approach—and one had documented clinical hip fusion), class 3 (two patients), and class 2 (one patient) (Figure 2, Figure 3 and Figure 4, Table 2). According to the Brooker classification [37], Brooker class 1 represents sporadic islands of radiographic calcifications, while class 2 represents obvious peri-trochanteric and pelvic HO, maintaining a minimum gap of 1 cm between the opposing bone surfaces. Class 3 is the same as class 2, with less than 1 cm between the opposing bone surfaces, and class 4 represents a radiographically fused (ankylosed) hip. HO started to appear as early as the second week on the postoperative radiographs and progressed throughout the follow-up. Notably, none of the patients received anti-HO prophylaxis postoperatively.

## 4. Discussion

This retrospective study includes six patients with cerebral palsy who had fractures of the neck of femur neck and were treated with hip hemiarthroplasty. The patients had a mean radiographic follow-up of 71.3 months. A significant finding in this cohort was the development of heterotopic ossification in all the patients. Ossification was severe enough to cause radiographic hip fusion (grade 4) in three out of six patients. Despite the small sample size, and subsequently the limited evidence, the 100% occurrence rate of HO and the overall guarded outcomes in our study may underscore the necessity of a personalized medical strategy in the surgical treatment of femoral neck fractures among people with cerebral palsy.

### 4.1. Pathophysiology of HO

Lamellar bone formation in tissues where bone typically does not exist is known as heterotopic ossification (HO). It is well-documented that HO can arise following hip replacement, often without symptoms [37,38]. Although the exact cause of HO is not fully understood, certain risk factors have been identified, including the male gender, burns, stroke, spinal cord injury (SCI), traumatic and inflammatory brain injury, previous HO history, ankylosing spondylitis, previous hip surgery, hypertrophic osteoarthritis, lateral and anterolateral hip approaches, Paget’s disease, diffuse idiopathic skeletal hyperostosis, myelopathies, rheumatoid arthritis, and Parkinson’s disease [37,38,39,40,41,42,43,44,45]. Cerebral palsy involves significant existing brain injury, which when combined with trauma-related triggers and altered bone metabolism in patients with CP, makes neurogenic HO likely to develop in patients with CP [41,44,46,47].

### 4.2. Personalized Medicine Approach

Based on the type and severity of neurological impairment in CP, a personalized medicine approach would allow for the customization of perioperative and surgical approaches for patients with CP who suffer from displaced femoral neck fractures. Information regarding the Gross Motor Function Classification System (GMFCS) of patient [48], the geographical CP distribution (hemiplegia, diplegia, or quadriplegic), the motor type (spastic, athetoid, or dystonic), the degree of hip pathology, and the concordance between the side affected by CP and the side of the hip that is involved [49] should be obtained. This information can guide the selection of surgical techniques (anterolateral approach versus posterior, with soft tissue repair), the choice of implant (i.e., a more constrained implant), and the development of targeted rehabilitation regimens (that focus on improving proprioception, regaining balance, anti-fall measures, range-of-motion exercises, and hip abductor strengthening) [50]. Surgeons ought to categorize patients with CP into precision-based groups, while considering any associated comorbidities when evaluating the option of arthroplasty.

### 4.3. Hip Arthroplasty in CP: Total Hip Arthroplasty

Multiple research studies have examined the THA results in individuals with CP. In a systematic review [51] comprising 21 studies (4886 THAs), the overall findings were favorable in terms of reducing pain and enhancing function. The most common complications reported included dislocation (with a mean incidence of 7.5% of patients, ranging from 0 to 28%), periprosthetic fractures (with a mean incidence of 5.6% of patients and a range from 0% to 21%), heterotopic ossification (present in 4.2% of patients, with a wide range from 0% to 37.8%), surgical site infections (2.1% of patients, ranging from 0% to 16.6%), acetabular/femoral loosening (3.7% of patients, with a range from 0% to 15.4%), and the necessity for revision surgery (required by 8.8% of patients, with a range of 0–19%). The survival rates for primary THAs vary from 85% to 100% at 5 years and from 73% to 86% at 10 years. The researchers observed also that heterotrophic ossification predominantly impacted pediatric and early adolescent populations with THA [51].

Another systematic review that included 15 studies (2732 THAs) [52] showed significant improvement in functional outcomes after total hip arthroplasty (THA) for patients with cerebral palsy (CP). However, there were various complications in 10–45% of cases. The commonest reported complication was instability (range 1–20%), followed by aseptic loosening (range 0.74–20%), periprosthetic femoral fracture (range 1.69–10%), surgical site infection (0.3–16.6%), chronic pain (range 1.2–12%), bursitis (range 8.4–20%), pressure ulcer (6.2–8.3%), and urinary tract problems (0.2–3.48%). The review found an incidence of heterotopic ossification (HO) of 2.5–53% and an 84% 10-year implant survival rate (range 81–86%) [52]. The two studies [51,52] did not consider HO a major concern because its incidence was like the reported HO incidence in hip surgeries in general, which can reach 53% [39]. Conversely, despite the 100% incidence of HO in our study, it should be noted that our study discussed the outcomes after HA in male patients with hip fractures exclusively, and the presence of trauma and the male sex are known risk factors for HO development [53,54,55].

### 4.4. Hip Hemiarthroplasty in CP: Literature Gap

Hip hemiarthroplasty continues to be a widely used treatment option for displaced femoral neck fractures in older populations and low-demand patients, and several studies have reported their successful outcomes in patients with different conditions [56,57,58,59,60]. The setting for hemiarthroplasty, however, differs from that of THA in terms of trauma presence, patient characteristics, activity level, and surgical settings, which may justify the discrepancies in complications risk between HA and THA [61,62].

Despite the abundance of studies that report the outcomes of THA in patients with CP, the existing evidence regarding HA in CP is still lacking, with only two case reports existing. Reis [26] reported on two patients with cerebral palsy HA who had intellectual impairment. The patients had repeated instances of instability and underwent surgical treatment involving the use of a bone block allograft to augment the posterior acetabulum, which led to the restoration of hip function and the prevention of further dislocations. Hiragami et al. [27] discussed a case of early dislocation following HA in a female patient with CP that required conversion to THA. Neither study reported the HO occurrence. In our study, the HO incidence is 100%, with severe HO developing in five out of the six cases, causing hip fusion in three, and potentially impacting the outcomes. As for the other complications in our study, one patient (16.67%) experienced an early postoperative dislocation, and another patient (16.67%) developed an acetabular protrusion, although the presence of grade 4 HO in this patient may have limited further acetabular protrusion.

The lack of evidence regarding HA in CP, while several studies exist on THA, may be attributed to several factors, such as the traditional perception of THA as the definitive intervention for hip fractures in younger patients and a surgical preference toward more restorative procedures in active individuals. A research bias toward studying populations with higher functional demands and/or better outcomes may also have contributed to this, since HA in patients with CP may be under-reported due to modest outcomes or higher complication rates. Our study aims to address this gap by focusing specifically on this understudied subgroup, reporting the outcomes, and advocating for a more individualized treatment approach.

### 4.5. Recommendations for HO Prophylaxis

Based on the findings of this study, it may be reasonable to consider using preventive measures against heterotopic ossification (HO) for patients with hip fractures and CP undergoing HA or other surgical procedures to treat hip fractures . These methods include the use of chemoprophylactic drugs postoperatively, which have been utilized with extensive dosage variations in the literature [63,64,65]. For example, Indomethacin (75 mg daily for 3 weeks), a selective COX-2 inhibitor (such as a 4-week course of Rofecoxib 25 mg per day), and Etidronate (20 mg/kg/day for 3 months followed by 10 mg/kg/day for an additional three months) have been mentioned, among other drugs and different doses, as effective measures for HO chemoprophylaxis [63,64,65]. Alternatively, the use of a single dose of from 700 to 800 Gy (centi-gray) of external beam irradiation within 72 h postoperatively has also been mentioned for HO prophylaxis [66]. Implementing any of these preventive measures seems like a reasonable step for CP hip HA patients to avoid the occurrence of HO based on the results of our series. It should be noted, however, that given the limited evidence supported by our small series, our hypotheses regarding HO prophylaxis in this patient setting warrant further investigation in future studies.

### 4.6. Physician-Patient Communication

Another important issue, given the overall guarded outcomes in this series, is the necessity of involving the patients as appropriate and caregivers in decision making. Sensibly presenting and discussing the potential outcomes is necessary to achieve realistic expectations.

## 5. Limitations

This study has several limitations. The low (level 4) evidence provided by this retrospective, non-comparative study is a major limitation that necessitates the careful interpretation of the findings. This study still cannot advocate for or oppose HA in patients with CP, and the retrospective nature by itself presents a major source of bias. The small number of cases, despite the rarity of this patient setting, is also a major limitation that further limits the generalizability of recommendations. The small sample size also largely limits the utilization of meaningful statistical analysis, which rendered the study hypotheses generated, rather than providing strong evidence-based outcomes. The other important issues are the lack of preoperative functional scoring and the existence of potential confounding factors, such as the chosen surgical approach, the patients’ demographics, and medical and cognitive comorbidities, which could all impact the interpretation of the outcomes and the incidence of complications, respectively. Despite all these limitations, this is the first and largest case series of HA in patients with CP, and it could open the door for better-evidenced studies. Ideally, a comparative, multicenter, large study would be required to elicit stronger evidence.

## 6. Conclusions

Considering the preliminary evidence presented in this study, hip hemiarthroplasty in patients with cerebral palsy may be associated with the overall guarded outcomes. Given the outcomes and the seemingly high incidence of heterotopic ossification in such a patient setting, the implementation of a precision medicine approach regarding decision making, the surgical approach, the postoperative rehabilitation protocols, and particularly possible prophylaxis against heterotopic ossification with drugs or radiotherapy is strongly supported by our data. The outcomes of hip hemiarthroplasty should be well-explained to patients with CP and their caregivers before surgery to achieve satisfactory outcomes. Future research should include larger series and possibly comparative studies to generate stronger evidence.

## Figures and Tables

**Table 1:** Medical and neurological comorbidities in patient cohort.

Comorbidities	Number of Patients
Intellectual disabilities	5
Seizures	5
Chronic kidney disease	2
Essential tremors	2
Heart failure	1

**Table 2:** Summary of cases and outcomes.

Case	Age	CP Type	Implant Type	Complications	Outcome
1	71	Ataxic	Cemented bipolar		HO grade 4
2	55	Quadriparitic	Cemented bipolar	Dislocation and early revision due to stem loosening. Recurrent dislocation managed with closed reduction and bracing.	Ho grade 3
3	68	Ataxic	Cementless unipolar		HO grade 4
4	60	Unidentified	Cemented bipolar		HO grade 2
5	33	Contralateral spastic hemiplegic	Cemented bipolar		HO grade 3
6	47	Ipsilateral spastic hemiplegic	Cementless bipolar	Acetabular protrusion, wheelchair bound	HO grade 4

## Data Availability

The data presented in this study are available on request from the corresponding author after completing a proper data use agreement document and securing IRB approval.

## Supplementary Materials

The following supporting information can be downloaded at: https://www.mdpi.com/article/10.3390/jpm15060252/s1, Table S1: STROBE Statement—checklist of items that should be included in reports of observational studies.
