# Supplementary material for: Guarded Outcomes After Hip Hemiarthroplasty in Patients with Cerebral Palsy: Highlighting a Personalized Medicine Approach to Mitigate the Risk of Complications
Source: J Pers Med. 2025 Jun 15;15(6):252. doi: 10.3390/jpm15060252 (PMC12194685; doi:10.3390/jpm15060252)
Supplement: Supplementary file 1 [file jpm-15-00252-s001.zip › jpm-3601481-supplementary.pdf]

Table S1. STROBE Statement—checklist of items that should be included in reports of observational studies

	Item No.	Recommendation	Page No.	Relevant text from manuscript
**Title and abstract**	1	(*a*) Indicate the study's design with a commonly used term in the title or the abstract	1	Abstract, lines 3 and 4
		(*b*) Provide in the abstract an informative and balanced summary of what was done and what was found	1	Abstract, methods and results.
**Introduction**				
Background/rationale	2	Explain the scientific background and rationale for the investigation being reported	2	Introduction, 1st-5th paragraphs.
Objectives	3	State specific objectives, including any prespecified hypotheses	2	Introduction, 5th paragraph.
**Methods**				
Study design	4	Present key elements of study design early in the paper	3	Materials and Methods: 2.1 Data acquisition section.
Setting	5	Describe the setting, locations, and relevant dates, including periods of recruitment, exposure, follow-up, and data collection	3	Materials and Methods: 2.1 Data acquisition and 2.2 Patients sections.
Participants	6	(*a*) *Cohort study*—Give the eligibility criteria, and the sources and methods of selection of participants. Describe methods of follow-up<br>*Case-control study*—Give the eligibility criteria, and the sources and methods of case ascertainment and control selection. Give the rationale for the choice of cases and controls<br>*Cross-sectional study*—Give the eligibility criteria, and the sources and methods of selection of participants	3	Materials and Methods: 2.1 Data acquisition and 2.2 Patients sections.
		(*b*) *Cohort study*—For matched studies, give matching criteria and number of exposed and unexposed<br>*Case-control study*—For matched studies, give matching criteria and the number of controls per case	/	/
Variables	7	Clearly define all outcomes, exposures, predictors, potential confounders, and effect modifiers. Give diagnostic criteria, if applicable	3	Materials and Methods: 2.1 Data acquisition and 2.2 Patients sections
Data sources/	8*	For each variable of interest, give sources of data and details of methods of assessment	3−5	Materials and Methods

measurement		(measurement). Describe comparability of assessment methods if there is more than one group		
Bias	9	Describe any efforts to address potential sources of bias	10	Limitations
Study size	10	Explain how the study size was arrived at	3	Sections 2.1 and 2.2
Quantitative variables	11	Explain how quantitative variables were handled in the analyses. If applicable, describe which groupings were chosen and why	3-5	Materials and Methods
Statistical methods	12	(*a*) Describe all statistical methods, including those used to control for confounding	5	Section 2.5
		(*b*) Describe any methods used to examine subgroups and interactions	5	Section 2.5
		(*c*) Explain how missing data were addressed	/	No missing data were recorded for the mini-series.
		(*d*) *Cohort study*—If applicable, explain how loss to follow-up was addressed<br>*Case-control study*—If applicable, explain how matching of cases and controls was addressed<br>*Cross-sectional study*—If applicable, describe analytical methods taking account of sampling strategy	/	No loss of follow up was recorded for the mini-series.
		(*e*) Describe any sensitivity analyses	/	No sensitivity analyses were performed in this mini-series
**Results**				
Participants	13*	(a) Report numbers of individuals at each stage of study—eg numbers potentially eligible, examined for eligibility, confirmed eligible, included in the study, completing follow-up, and analysed	5	section 3: Results
		(b) Give reasons for non-participation at each stage	/	All relevant subjects were included in the study
		(c) Consider use of a flow diagram	7	A table summarizing the included patients was added (Table 2)
Descriptive data	14*	(a) Give characteristics of study participants (eg demographic, clinical, social) and information on exposures and potential confounders	5-7	Section 3: Results
		(b) Indicate number of participants with missing data for each variable of interest	7	Table 2
		(c) *Cohort study*—Summarise follow-up time (eg, average and total amount)	5	Section 3: Results
Outcome data	15*	*Cohort study*—Report numbers of outcome events or summary measures over time	5-8	Section 3: Results<br>Section 4. Discussion
		Case-control study—Report numbers in each exposure category, or summary measures of	/	/

		exposure		
		Cross-sectional study—Report numbers of outcome events or summary measures	/	/
Main results	16	(*a*) Give unadjusted estimates and, if applicable, confounder-adjusted estimates and their precision (eg, 95% confidence interval). Make clear which confounders were adjusted for and why they were included	5-8	Applicable estimates were provided.
		(*b*) Report category boundaries when continuous variables were categorized	/	/
		(*c*) If relevant, consider translating estimates of relative risk into absolute risk for a meaningful time period	/	/
Other analyses	17	Report other analyses done—eg analyses of subgroups and interactions, and sensitivity analyses	/	No other analyses performed.
**Discussion**				
Key results	18	Summarise key results with reference to study objectives	8	Section 4, first paragraph
Limitations	19	Discuss limitations of the study, taking into account sources of potential bias or imprecision. Discuss both direction and magnitude of any potential bias	10-11	Section 5: Limitations
Interpretation	20	Give a cautious overall interpretation of results considering objectives, limitations, multiplicity of analyses, results from similar studies, and other relevant evidence	11	Section 6: Conclusions
Generalisability	21	Discuss the generalisability (external validity) of the study results	10-11	Section 5: Limitations
**Other information**				
Funding	22	Give the source of funding and the role of the funders for the present study and, if applicable, for the original study on which the present article is based	11	No funding received

*Give information separately for cases and controls in case-control studies and, if applicable, for exposed and unexposed groups in cohort and cross-sectional studies.

Note: An Explanation and Elaboration article discusses each checklist item and gives methodological background and published examples of transparent reporting. The STROBE checklist is best used in conjunction with this article (freely available on the Web sites of PLoS Medicine at http://www.plosmedicine.org/, Annals of Internal Medicine at http://www.annals.org/, and Epidemiology at http://www.epidem.com/). Information on the STROBE Initiative is available at www.strobe-statement.org.
